# Genetic and Epigenetic Regulation of MEFV Gene and Their Impact on Clinical Outcome in Auto-Inflammatory Familial Mediterranean Fever Patients

**DOI:** 10.3390/cimb45010048

**Published:** 2023-01-13

**Authors:** May E. Zekry, Al-Aliaa M. Sallam, Sherihan G. AbdelHamid, Waheba A. Zarouk, Hala T. El-Bassyouni, Hala O. El-Mesallamy

**Affiliations:** 1 Molecular Genetics and Enzymology Department, Human Genetics and Genome Research Division, National Research Centre, Cairo 12622, Egypt; 2 Biochemistry Department, Faculty of Pharmacy, Ain Shams University, Cairo 11566, Egypt; 3 Clinical Genetics Department, National Research Centre, Cairo 12622, Egypt; 4 Dean of Faculty of Pharmacy, Sinai University, North Sinai 45518, Egypt

**Keywords:** familial Mediterranean fever, epigenetics, methylation, pyrin, colchicine, MEFV gene

## Abstract

Epigenetic modifications play a pivotal role in autoimmune/inflammatory disorders and could establish a bridge between personalized medicine and disease epidemiological contexts. We sought to investigate the role of epigenetic modifications beside genetic alterations in the MEFV gene in familial Mediterranean fever (FMF). The study comprised 63 FMF patients diagnosed according to the Tel Hashomer criteria: 37 (58.7%) colchicine-responders, 26 (41.3%) non-responders, and 19 matched healthy controls. MEFV mutations were detected using a CE/IVD-labeled 4-230 FMF strip assay. DNA methylation of MEFV gene exon 2 was measured using bisulfite modification and related to pyrin level, phenotypic picture, MEFV mutations, disease severity, serum amyloid A (SAA), CRP, ESR, disease severity, and colchicine response. Our results showed that FMF patients exhibited significantly higher methylation percentage (*p* < 0.001) and lower pyrin levels (*p* < 0.001) compared to the control. The MEFV gene M694I mutation was the most commonly reported mutation (*p* < 0.004). High methylation percentage of the MEFV exon 2 and low pyrin concentration were correlated with disease severity, high SAA, ESR levels, H-pylori, and renal calculi. In conclusion, this study highlights the relation between high methylation percentage, reduced pyrin level, and different biomarkers in FMF, which underscores their role in the pathogenesis of FMF and could be considered as potential therapeutic targets.

## 1. Introduction

Epigenetics is the study of a diverse array of reversible, dynamic, and heritable phenotypic alterations to the genome that affect gene expression without affecting genomic DNA sequence [1]. These epigenetic modifications can be inherited through generations and show inter-individual variations [2]. They play a role in human evolution and adaptation [3], and cell differentiation and specification by regulating gene expression and silencing in a context-dependent manner [4]. Epigenetic deregulation has been studied in the pathogenesis of various diseases including cancer, cognitive disability, COVID-19 severity, neurodevelopmental, neurodegenerative, autoimmune, and auto-inflammatory disorders [5,6,7].

Epigenetic mechanisms include DNA methylation, histone modifications, non-coding RNAs, and chromatin remodeling [2]. DNA methylation is considered the most common epigenetic mechanism, which involves the transfer of a methyl group from S-adenosyl-L-methionine (SAM) through the catalytic action of DNA methyl-transferases (DNMTs) to the fifth carbon atom of cytosine residues (5mC) on CPG-islands [8]. It has been shown that DNMTs perform various functions, where DNMT1 is involved in cell division [8], DNMT3a establishes maternal imprints in differentially methylated regions, and DNMT3b is required for X chromosomes inactivation [8]. On the other hand, DNA de-methylation can occur by actively reversing DNA methylation through oxidation of the 5mC at methylated CPG sites to 5-hydroxymethylcytosine, 5-formylcytosine, or 5-carboxylcytosine that is catalyzed by the ten-eleven translocation family members and thymine DNA glycosylase enzymes, increasing the transcriptional activity of the genes [9].

DNA methylation influences a wide variety of biological processes such as gene regulation, expression, imprinting, chromatin configuration, DNA structural stability, and transcriptional silencing through inhibition of the elongation of RNA polymerase 2 transcription [10], repression of gene activity by inhibiting the binding of transcription factors to DNA [11], and recruiting methyl-DNA binding domain proteins (MBDs) [5,12]. Additionally, it plays a pivotal role in the gene–environment interaction and their impact on the pathogenesis of various diseases [13,14].

Furthermore, DNA methylation is one of the key regulators in immune cell differentiation and development [8,9]. It plays an important role in macrophage, T-cell, and B-cell development, function, regulation, differentiation, and memory [8,9]. In addition, it is involved in NK cell differentiation, where hypo-methylation of promoter regions of killer Ig-like receptor and CD94/NK group 2 member A results in NK cells’ maturation and transcription up-regulation [9]. DNA methylation could reduce gene expression by either blocking initiation sites of transcription or interacting with nucleosomes that lead to hetero-chromatinization [10,15]. On the other hand, DNA methylation could enhance gene expression through alternative promoters or by blocking insulators [10]. In addition, the DNA binding capacity of many transcription factors is affected by the methylation of their binding sequences [16]. Although hyper-methylation of CpG islands, particularly in the promoter region, is highly correlated with transcriptional silencing [10,15,17], it was also reported that methylation in the body of genes acts as a positive regulator of transcription [10].

Deregulation in DNA methylation is considered as a biomarker for early detection and risk stratification of various autoimmune diseases including systemic lupus erythematosus, type 1 diabetes, rheumatoid arthritis, Graves’ disease, and Hashimoto’s disease [8,18].

Familial Mediterranean fever (FMF) is the most common monogenic auto-inflammatory disease, affecting mainly the Mediterranean races [19] with a high prevalence in the Egyptian population [20]. It is characterized by recurrent fever attacks, erysipelas-like erythema, peritonitis, arthritis, and pleurisy, with amyloidosis as the major long-term complication [21,22]. Colchicine is the mainstay of FMF treatment, which is safe and effective in preventing FMF clinical attacks and secondary amyloidosis [23].

FMF is caused by mutations of the MEFV gene located on 16p13.3, composed of ten exons, with the 998 bp CpG island covering the second exon [24]. It encodes the pyrin protein, which plays an important role in innate immune responses and in cyto-skeletal signaling pathways [10,25], where it can sense intracellular danger signals and activate caspase-1, which in turns activates nuclear factor-kappa B and results in the maturation of IL-1β and IL-18 [10]. Thus, pyrin can inhibit the inflammatory response by stimulating the inhibition of pro-inflammatory molecules and transcription of anti-inflammatory proteins [10]. 

Several studies have addressed disease-causing mutations in FMF particularly at the carboxy-terminal B30.2 domain portion of pyrin [24,25,26], that can result in either loss of function or gain of function [19]. Some mutations can result in gain-of-function with subsequent increase in pyrin expression in FMF patients [26,27], while other mutations can result in pyrin knockdown resulting in increased IL-1β release and caspase-1 activation [28]. C-terminal B30.2 domain’s heterozygote variants accentuate pyrin activity, while heterozygote variants in the other pyrin domains affect residues essential for inhibition or protein oligomerization, leading to active inflammasome [29]. MEFV mutations at the 10th exon (M694V, M680I, and V726A) can reduce the interaction between the B30.2 domain and caspase-1, causing caspase-1 inhibition, IL-1β release, reduced pyrin activity, abnormal inflammasome function, and the inflammatory reactions [30]. This controversy in the impact of genetic mutations on pyrin level may depend on their association with epigenetic factors which could, in turn, shape disease presence and severity [5]. 

MEFV gene methylation and pyrin levels are considered as potential factors in the FMF pathogenesis [10], with previous studies showing controversial results [10,31,32]. Methylation is involved in the FMF pathogenesis through MEFV exon 2 splicing and protein aberrant localization, irrespective of the presence of the MEFV pathogenic variants [15,16]. The fully spliced MEFV second exon shows the highest expression, in comparison to the full-length exon [15,16,33], as the majority of the CpG-islands of 568 bp (41/66) are spanning MEFV gene complete exon 2 [33].

In this respect, we sought to investigate the role of methylation% of the MEFV gene exon 2 as a biomarker for FMF in the Egyptian population, and whether DNA methylation could be used as a diagnostic and prognostic marker by studying its relation to pyrin level, disease severity, phenotypic picture, MEFV gene mutations, and colchicine response. Moreover, we emphasize the pharmaco-epigenetics and the pharmaco-genetics in colchicine non-responsive FMF patients.

## 2. Materials and Methods

### 2.1. Patients

A total of 63 patients and 19 healthy controls were recruited from the Clinical Genetics Clinic, National Research Centre, Egypt. This study was approved by the Local Research Ethics Committee of the Faculty of Pharmacy, Ain-Shams University (Number 80, 24 October 2018). Written informed consent was obtained from the parents of all participants in the study. 

All FMF patients were diagnosed based on the Tel Hashomer clinical criteria [33]. They were classified into colchicine-responders (n = 37, 58.7%), represented by FMF patients with complete response to colchicine treatment whose episodes were completely recovered by the standard dose of colchicine treatment and without any episode in the previous year, and non-responders (n = 26, 41.3%), represented by FMF patients with no response to colchicine treatment whose episode frequency was not decreased despite the standard colchicine treatment and having more than 1 attack every 3 months.

Demographic data, frequency of the attacks, response to treatment, complete blood count (CBC), erythrocyte sedimentation rate (ESR), C-reactive protein (CRP), kidney function, serum amyloid A (SAA), urine analysis, and Helicobacter pylori (H-pylori) laboratory tests, as well as abdominal and pelvic ultrasonography were extracted from the medical records. 

#### Disease Severity

Disease severity was calculated based on the Pras severity scoring system for pediatric FMF [34], and patients were divided into 3 subgroups according to this score: patients with mild disease (score between 3–5), patients with moderate disease (score between 6–8), and patients with severe disease (score ≥ 9). Disease severity correlates with early-onset disease, causing early admission, number of attacks, acute or degenerative arthritis, and presence of erysipelas-like erythema, amyloidosis, and colchicine dose.

### 2.2. Methods

#### 2.2.1. Blood Sample Collection

Venous blood samples (2 mL) were collected in EDTA anti-coagulated vacuum tubes for mutation, methylation%, and pyrin concentration analyses.

#### 2.2.2. Mutation Analysis

A CE/IVD-labeled 4-230 FMF Strip Assay (Vienna Lab Diagnostics, Vienna, Austria) was used for detecting the 12 most common MEFV mutations in the Mediterranean region, which were Exon 2 E148Q and R202Q; Exon 3 P369S; Exon 5 F479L; Exon 10 R761H, I692del, M680I, M694V, M694I, K695R, V726A, and A744S.

#### 2.2.3. Methylation Analysis

An EZ DNA Methylation Gold Kit (Zymo Research, Irvine, CA, USA) D5030 was used to perform DNA bisulfite modification. An ABI 7500 Real Time PCR System (Applied Bio Systems, Roche Light Cycler) and HERA SYBR Green qPCR Kit (Willow Fort) were used with the following protocol: pre-incubation at 95 °C for 10 min, denaturation at 95 °C for 30 s, annealing at 50 °C for 60 s, then extension at 72 °C for 60 s, repeated for 40 cycles, final extension at 72 °C for 7 min, and cooling at 4 °C for >5 min. One set of primer sequences that was designed for the methylated form of the DNA sequence was:

Forward primers TAAGCAACTTGGGTTTGCCATTC

Reverse primers TAAGGCCCAGTGTGTCCAAGTG 

while primer sequences designed for the un-methylated form of the DNA sequence were: 

Forward primers TCTGTGTAAGCAACTTGGGTTTG

Reverse primers GTAAGGCCCAGTGTGTCCAAGT

Then, the methylation% of the MEFV gene exon 2 was calculated as previously described [35,36]. Percent methylation = 100 × 2^−ΔCt^, where ΔCt = the average Ct value from the test reaction minus the average Ct values from the reference reaction.

#### 2.2.4. Pyrin Analysis

A commercial sandwich ELISA kit obtained from Sinogeneclon Biotech Co., Hangzhou, China Ltd. Catalog No: SG-12990 was used for the quantitative determination of human pyrin concentration, and spectro-photometric measurements were performed on Thermo Scientific (Waltham, MA, USA) Multiskan GO ELISA plate reader for pyrin concentration calculations.

#### 2.2.5. Receiver Operating Characteristic Curve (ROC Curve)

The diagnostic potential of the methylation% of the MEFV gene exon 2 and the pyrin level in FMF patients were assessed using a ROC curve to detect their sensitivity and specificity.

### 2.3. Statistical Analysis

SPSS Windows application was used to conduct all statistical analysis (version 20.0; SPSS Inc., Chicago, IL, USA). The quantitative variables were defined in the form of means and standard deviations (SDs), using the Shapiro–Wilk normality tests for their comparison. Whenever the test results showed that the data were normally distributed, the independent Student’s *t*-test (parametric tests) and one-way ANOVA test were used; the categorical variables were tested using the chi-square test. When the data were not normally distributed (non-parametric tests), the Mann–Whitney test was used. *p* < 0.05 was significant for comparisons.

## 3. Results

### 3.1. Demographic Data

As depicted in Table 1, the study comprised 63 FMF patients (with a female predominance of 35 female: 28 male (1.25:1), age 9.7 ± 4.4 years, age at disease onset 5.3 ± 3.7 years, and receiving colchicine dose ranging from 0.5 to 2.5 mg/day; and 19 healthy controls (9 males and 10 females, age 5–20 years). There were 22 patients who were consanguineous marriage offspring (34.9%) and 54 patients (85.7%) were similarly affected family members.

### 3.2. Methylation% and Pyrin Level

**Figure 1:**
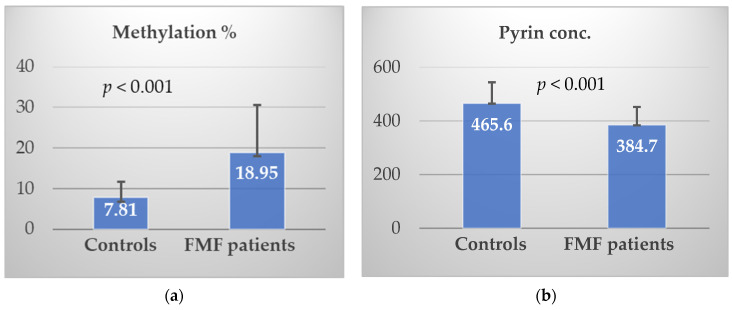
(**a**) Distribution of the MEFV exon 2 methylation% among FMF patients and controls; (**b**) distribution of the pyrin level among FMF patients and controls.

Our results showed that the methylation% of the MEFV exon 2 in Egyptian FMF patients (18.94 ± 1.46) was significantly higher than that in the control group (7.81 ± 0.87) (*p* < 0.001 **), Figure 1a. While the pyrin concentration (pg/mL) in FMF patients (384.72 ± 67.44) was significantly lower than that in the control group (465.55 ± 78.41) (*p* < 0.001 **), Figure 1b.

**Figure 2:**
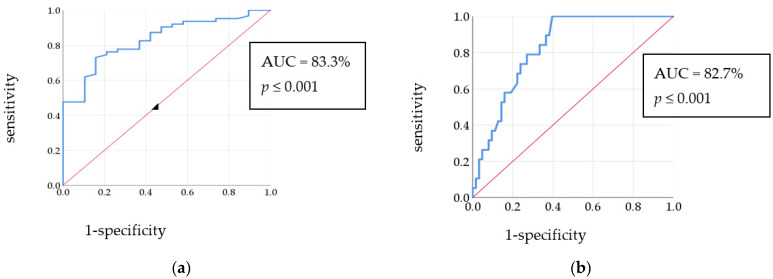
(**a**) ROC curve for the methylation% of MEFV gene exon 2 in FMF patients and control group; (**b**) ROC curve for the pyrin level in FMF patients and control group.

Concerning the ROC curve for the methylation% of the MEFV gene exon 2, the area under the curve (AUC) was 83.3%. Coordinates of the curve showed that the cut-off value was ≥10.41%, which predicted FMF with a sensitivity of 73.0% and specificity of 84.2%, Figure 2a. In the ROC curve for pyrin concentration in FMF patients and the control group, the AUC was 82.7% and the cut-off value was ≥393.35 pg/mL with 100.0% sensitivity and 60.3% specificity, Figure 2b.

**Figure 3:**
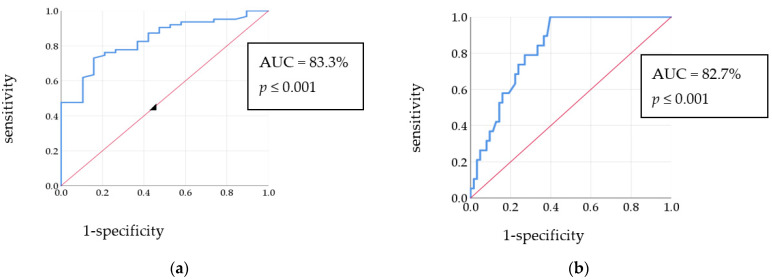
Correlation of the MEFV exon 2 methylation% and the pyrin concentration among: (**a**) controls; (**b**) FMF patients.

The correlation between the mean methylation% of the MEFV exon 2 and that of pyrin concentration was significantly positive in the control group (r = 0.584, *p* = 0.009 **) and non-significant among FMF patients (r = 0.01, *p* = 0.938), Figure 3.

Regarding the mean methylation% of the MEFV exon 2 and the pyrin concentration, no statistically significant difference was observed between male and female or between similarly and non-similarly affected family member FMF patients. On the other hand, the consanguineous marriage offspring showed a significant higher methylation% of the MEFV exon 2 than the non-consanguineous marriage offspring. Moreover, no statistically significant difference was detected in the pyrin concentration in the two subgroups, Table 2.

### 3.3. Genotyping

The MEFV gene mutations were detected in 49 out of 63 FMF patients (77.8%), and not detected in healthy controls. As shown in Table 3, a statistically significant difference was observed between FMF patients with and without the MEFV gene mutations (*p* = 0.001 **), those with different detected MEFV mutations (*p* = 0.004 **), and those with different MEFV status (*p* = 0.001 **). Moreover, the FMF patients with the MEFV gene exon 10 mutations were significantly more than those with exon 2 mutations detected (*p* ≤ 0.001), Table 3.

M694I mutation (n = 19, 30.2%) was the most commonly reported, followed by M680I (n = 13, 20.6%), E148Q (n = 11, 17.5%), V726A (n = 6, 9.5%), M694V (n = 4, 6.3%), and R202Q (n = 2, 3.2%). The most common genotype was heterozygous (n = 34, 53.96%), followed by homozygous (n = 8, 12.6%), and compound heterozygous (n = 7, 5.55%).

On the other hand, no significant differences were detected concerning the association of the MEFV exon 2 methylation% and the pyrin level with genotyping among FMF patients, except the MEFV exon 2 methylation% of homozygous M694I (37.205 ± 6.6), which was significantly higher than homozygous M694V (22.48 ± 5.6) and M680I (9.88 ± 1.2) (*p* = 0.028), Table 3.

The MEFV exon 2 methylation% in FMF patients harboring different mutations was as follows: R202Q (25.1 ± 20.8), M694V (24.8 ± 9.3), M694I (21.5 ± 11.9), E148Q (20.9 ± 15.7), V726A (18.9 ± 7.9), and M680I (18.9 ± 7.9) (*p* = 0.448), while the highest pyrin concentration (pg/mL) was detected in FMF patients who carried M694V (416.8 ± 60.9), followed by V726A (399.5 ± 52.1), M680I (395.6 ± 56.7), M694I (387.2 ± 88.8), E148Q (366.9 ± 63.1), and R202Q (322.3 ± 36.8) mutations (*p* = 0.601).

### 3.4. Disease Severity and Studied Parameters

According to the PRAS severity scoring system, a statistically significant difference was detected between the frequency of mild (13 (20.6%)), moderate (18 (28.6%)), and severe (32 (50.8%)) FMF patients (*p* ≤ 0.001 **). 

The incidence of similarly affected family members in patients with severe disease was (28/32, 87.5%), moderate disease (18/18, 100%) and mild disease (8/13, 61.5%) (*p* = 0.01 **). The majority of patients with mild FMF severity were offspring of non-consanguineous marriage (12 patients, 92.3%, *p* = 0.02 *).

Additionally, the methylation% of the MEFV exon 2 in severe and moderate patients was significantly higher than that in mild FMF patients (*p* = 0.016 *), while the pyrin concentration in severe cases was non-significantly lower than that in moderate and mild patients (*p* = 0.664), Table 4.

No statistically significant difference was detected between severity and genotype, except patients with the homozygous M694V mutation suffered from the severe form of the disease (*p* = 0.042), while those with the M680I mutation were mostly associated with the moderate disease severity, Table 5.

### 3.5. Biomarkers

Elevated SAA (≥6.4 mg/L), CRP (>4 mg/L), and ESR (>10 mm/HR) levels were detected in 13 (20.6%), 21 (33.3%), and 28 (44.4%) FMF patients, respectively. In addition, 48.6% and 62.9% of female FMF patients exhibited high CRP and ESR compared to 14.3% and 21.4% of male FMF patients (*p* = 0.004 and 0.001, respectively). Moreover, we found that more than half of the studied patients suffer from anemia (32, 50.8%), 14 patients had renal calculi (22.2%), and 8 patients had H-pylori (12.7%).

No statistically significant difference was noted between MEFV exon 2 methylation% with high and normal markers: SAA (19.64 ± 12.8 vs. 8.77 ± 11.4, *p* = 0.852); ESR (21.42 ± 12.1 vs. 16.97 ± 10.9, *p* = 0.173); CRP (18.9 ± 11.4 vs. 18. ± 11.4); H-pylori (20.88 ± 17.6 vs. 18.67 ± 10.7, *p* = 0.71); anemia (20.88 ± 17.6 vs. 18.67 ± 10.7, *p* = 0.945); and vitamin D (20.7 ± 11.1 vs. 17.8 ± 11.9, *p* = 0.195) except for that of patients with renal calculi (25.12 ± 16), which was significantly higher than those without renal calculi (17.19 ± 9.4, *p* = 0.023 *).

Additionally, no statistically significant difference was observed between the pyrin concentration (pg/mL) in FMF patients with high and normal markers: SAA (372.86 ± 19.63 vs. 387.82 ± 8.5, *p* = 0.48); ESR (372.83 ± 12.28 vs. 394.25 ± 8.5, *p* = 0.21); CRP (393.0 ± 15.36 vs. 380.59 ± 70.7, *p* = 0.49); H-pylori (376.33 ± 75.0 vs. 385.95 ± 76.0, *p* = 0.70); anemia (388.16 ± 69.0 vs. 381.18 ± 66.8, *p* = 0.68); vitamin D (389.8 ± 12.86 vs. 381.55 ± 70.7, *p* = 0.63); and renal calculi (388.75 ± 88.4 vs. 383.58 ± 61.3, *p* = 0.80).

### 3.6. Phenotype and Studied Parameters

The most commonly reported FMF complaint was abdominal pain, followed by arthritis and fever. Moreover, the highest MEFV exon 2 methylation% was reported in patients suffering from vomiting, followed by erysipeloid erythema, diarrhea/constipation, abdominal pain, arthritis, fever, and chest pain, Table 6.

Furthermore, there was a significant difference between the methylation% in patients with vomiting and those without vomiting (17.76 ± 11.1) (*p* = 0.043). On the other hand, the highest pyrin concentration was reported in FMF patients suffering from chest pain followed by vomiting, fever, abdominal pain, erysipeloid erythema, arthritis, and diarrhea/constipation. Moreover, there was a significant difference between the pyrin concentration in FMF patients with chest pain and those without chest pain (378.75 ± 76.0) (*p* = 0.029).

No correlation was detected between the MEFV exon 2 methylation% or the pyrin concentration and patients with different phenotypes, Table 6.

### 3.7. Colchicine Response

#### 3.7.1. Colchicine Response among Patients

In total, 37 (58.7%) FMF patients responded to colchicine treatment, and 26 (41.3%) patients did not respond to colchicine treatment. All FMF clinical symptoms and severe disease were significantly more present in colchicine-non-responders than colchicine-responders, Table 7. No significant difference was detected between colchicine-responders and non-responders concerning genotyping (*p* = 0.628), Table 7. There was a significant difference between the distribution of colchicine-responders with homozygous and compound heterozygous mutations of M694I1 (14.3%), M694V2 (100.0%), M680I 3 (37.5%), and M726V (0) and the heterozygous versions of the same mutations (6 (85.7%), 0, 5 (62.5), and 4 (100.0%), respectively) (*p* = 0.05). Moreover, all the patients with mild disease were colchicine-responders in our study. High ESR and anemia were more significantly detected in colchicine-non-responders than colchicine-responders, Table 7.

#### 3.7.2. Colchicine Response, Methylation%, and Pyrin

The MEFV exon 2 methylation% mean in colchicine-non-responders was non-significantly slightly higher than responders, and the mean pyrin concentration in colchicine-non-responders was non-significantly lower than that of responders, Table 7.

**Figure 4:**
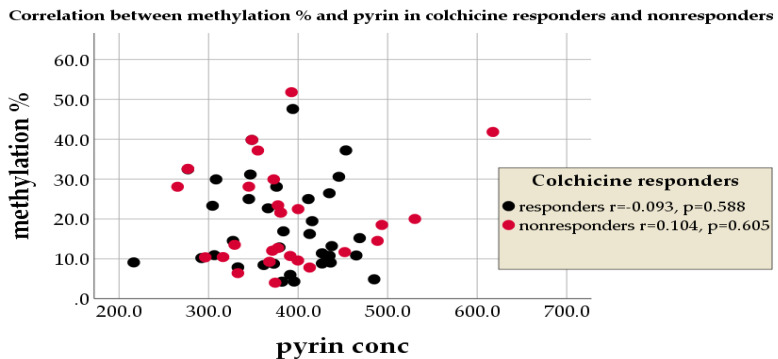
Correlation between the MEFV exon 2 methylation% and the pyrin concentration among FMF colchicine-responders and non-responders.

No significant negative correlation was noted between the MEFV exon 2 methylation% and pyrin concentration among FMF colchicine-responders (r = −0.093, *p* = 0.588), and a positive correlation was noted between them in non-responders (r = 0.141, *p* = 0.605), Figure 4.

### 3.8. Correlations between Studied Biomarkers

**Figure 5:**
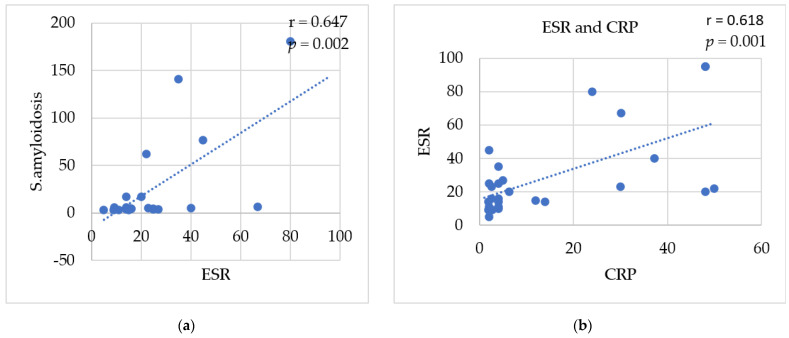

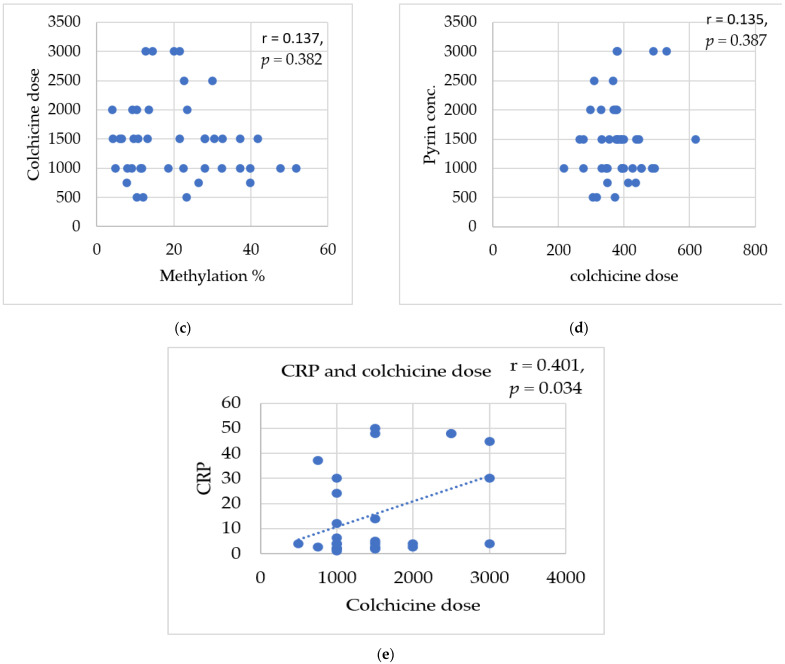
Correlation between biomarkers, colchicine dose, MEFV exon 2 methylation%, and pyrin level among FMF patients: (**a**) ESR and SAA; (**b**) ESR and CRP; (**c**) colchicine dose and MEFV exon 2 methylation%; (**d**) colchicine dose and pyrin level; (**e**) colchicine dose and CRP.

A significant positive correlation was detected between ESR and SAA levels (r = 0.647 and *p*-value = 0.002 **), Figure 5a, and between ESR and CRP levels (r = 0.618, *p* = 0.001 **), Figure 5b. Furthermore, a significant correlation was detected between colchicine dose (1470.93 mg/day ± 692.7) and CRP level (14.13 mg/L ± 17.4) (r = −0.401, *p* = 0.034 *), Figure 5e, while a non-significant correlation was detected between colchicine dose (1470.93 mg/day ± 692.7) and the MEFV exon 2 methylation% (18.95% ± 11.6) (r = −0.137, *p* = 0.382) and pyrin level (r = 0.135, *p* = 0.387) among the patients, Figure 5.

## 4. Discussion

The association of epigenetic modifications and autoimmune disorders has been an area of active research over the last decade [10]. Several studies have addressed FMF disease in the Egyptian population [20,37,38,39,40,41,42,43], but the novel portrayals in our study are detecting the methylation% of the MEFV exon 2 and the pyrin concentration in FMF Egyptian patients, and whether we could target DNA methylation and pyrin level as future diagnostic and prognostic biomarkers by studying their relations with gender, consanguineous marriage, similarly affected family members, phenotype, disease severity, genotype, biomarkers, and drug response. 

DNA methylation is a stable epigenetic modification that can be utilized as a biomarker in the clinical management and personalization of patient care in various diseases [44]. The methylation% of the MEFV exon 2 was significantly higher in FMF patients than controls in this Egyptian cohort. This corroborates the findings of a previous investigation [34], while others suggested that the methylation status of the exon 2 in the MEFV gene is non-significantly higher than healthy controls [10]. The difference may be due to differences in population, the role of ancient migrations, nutritional habits, and cultural traditions, as well as sample size [45,46,47,48]. Moreover, in our study, we detected the most common MEFV mutations in the Mediterranean region (Exon 2 E148Q and R202Q; Exon 3 P369S; Exon 5 F479L; Exon 10 R761H, I692del, M680I, M694V, M694I, K695R, V726A, and A744S), while Dogan et al. studied only the five most common mutations M694V, M680I, M694I, E148Q, and V726A, most of which were located on exon 10 [10].

DNA methylation influences a large variety of biological processes such as gene regulation, expression, and repression of gene activity [10,11]; therefore, we studied the pyrin concentration in FMF Egyptian patients and its correlation with the methylation% among the Egyptian population. Pyrin concentration was significantly lower in FMF Egyptian patients compared to the healthy controls. This is in accordance with a Turkish study revealing that the MEFV gene mutations lead to deficient levels of pyrin [10]. In contrast, another study highlighted that pyrin concentration is higher in FMF patients than controls [33]. Hence, the MEFV exon 2 methylation% and pyrin level may have a role in the pathogenesis of the FMF disease. Although significant positive correlation was observed between the methylation% of the MEFV exon 2 and that of pyrin concentration in the control group, no correlation was detected in FMF patients. Several factors can affect pyrin levels, including C-terminal B30.2 domain mutations, novel mutations, dysfunction of other genes regulating the gene expression, protein transport dysfunctions, environmental influences, and other epigenetic factors such as histone modification and miRNAs [10,26,27,28]. Histone alterations and CpG methylation are linked throughout MBD, inducing two histone de-acetylases and methyl-transferases that lead to silencing and prevent the DNMT3 linkage, which is important in methylation [12]. MiRNAs regulate gene expression at a post-transcriptional level by degrading mRNA molecules or blocking their translation [10]. Additionally, microorganisms may affect FMF since pyrin is a component of NLRP3, which is a pathogen-recognition receptor, and bacterial pathogens could change the phosphorylation status of pyrin and its activation [31].

Concerning the methylation effect on the clinical diversity and genotyping in FMF patients, the MEFV mutations profile is variable according to ethnicity [49]. The most common reported MEFV gene mutations are M694V, M726V, and M680I in Mediterranean populations [49,50]. Moreover, in our study, the most common mutations were M694I, M680I (G/C), E148Q, and V726A. This agrees with a previous Egyptian study [39]. On the other hand, other studies have reported the most common mutations among the Egyptian FMF patients were M694V in Delta governorates [37], E148Q [20,39,43], and V726A [41,42]. This diversity reflects the heterogeneity of the population and the heterogeneous origin of FMF in Egypt [51]. 

In the current study, MEFV exon 10 mutations were significantly more prevalent than exon 2 mutations. This coincides with previous studies observing that exon 10 mutations have been found in 91% of FMF patients [52]. Additionally, the pyrin concentration of patients with MEFV exon 2 mutations was non-significantly lower than that for exon 10 mutations. This corresponds with Neslihan et al. study reporting that increased pyrin protein levels in FMF patients could be independent of MEFV gene exon 10 mutations [32]. This discrepancy may be due to the transcriptional silencing of the methylation CpG-islands [10], as the mean methylation% of the MEFV exon 2 was non-significantly higher than that of the studied exon 10 mutations. This is in agreement with a previous study revealing that most of the CpG-islands are more concentrated in exon 2 than exon 10 [50].

In agreement with previous studies in the Egyptian population [38,40], the heterozygous genotype was more predominant in our study, followed by homozygous and compound heterozygous. Additionally, no significant difference was shown concerning the association of the MEFV exon 2 methylation% and the pyrin level with genotyping status among FMF patients, which agrees with previous studies [10,26] and may be due to the MEFV exon 2 splicing [15,16].

Regarding the patients’ clinical diversity, the most-reported complaint was abdominal pain, followed by arthritis and fever, which agrees with a previous study in the Egyptian population [20,40,43]. On the other hand, other studies have reported that arthritis [37] or fever [23] was the most common FMF complaint. This diversity corroborates the finding of previous investigations that phenotypic heterogeneity is very common in FMF patients [20,45], and might be due to other causes of abdominal pain such as irritable bowel syndrome, colchicine intolerance, and amyloid accumulation in the small intestine [44]. Moreover, we found a relation between the methylation level of the MEFV exon 2 and the phenotype, as demonstrated by FMF patients complaining of abdominal pain, arthritis, erysipeloid erythema, and constipation/diarrhea who had non-significant higher methylation% than those with fever and chest pain, while another study highlighted that there is no relation between the methylation% of exon 2 in the MEFV gene and clinical symptoms [10]. Furthermore, the association between the pyrin concentration and the phenotype was evidenced by the higher pyrin concentration in FMF patients complaining of chest pain, followed by vomiting, fever, abdominal pain, erysipeloid erythema, arthritis, and diarrhea/constipation. This contradiction in the pyrin concentration may be due to other factors affecting the FMF phenotype including environmental, epigenetic factors such as methylation and genetic factors such as the MEFV mutations and modifier genes [10]. 

Additionally, we found a direct relationship between the methylation% of the MEFV exon 2 and disease severity, but an inverse relationship between the pyrin concentration and disease severity. The FMF patients suffering from the moderate or severe form of the disease had a significantly higher MEFV exon 2 methylation% and non-significantly lower pyrin concentration than those with mild FMF severity. Therefore, methylation may contribute to the aggravation of the severity of the disease. No relation was detected between severity and mutations, except patients with the M694V mutation significantly suffered from the severe form of the disorder. This agrees with a previous Egyptian study [38], and may be due to MEFV-independent genetic modifiers such as IL1B polymorphism, other MEFV-independent modifying loci, or the major histocompatibility complex class I chain-related gene A (MICA) [53,54,55]. 

In this study, high SAA, ESR, and CRP levels were detected in 20.6%, 44.4%, and 33.3%, respectively, of the studied patients, while higher levels were reported in other Egyptian studies [39,56]. The methylation% of the MEFV exon 2 in FMF patients with renal calculi was significantly higher than those without renal calculi.

The overall phenotype of FMF patients was also likely to be influenced by non-genetic factors, such as gender. In parallel with a previous study [56], Egyptian FMF female predominance was observed, which is in contrast to other studies reporting significant predominance of the male gender in the FMF Egyptian population [20,51]. This may be related to the higher methylation% of the MEFV exon 2 in FMF females than males in our study. The pyrin concentration was similar in both genders. This diversity may be due to the fact that DNA methylation plays a role in X chromosome activation [57]. The methylation level of the MEFV exon 2 in patients with consanguineous parents was significantly higher than in those without consanguineous parents, underscoring that consanguineous marriage and similarly affected family members play an important role in FMF disease inheritance in Egypt [57]. 

All these diversities and contradictions may have a relation with the drug response among the FMF patients. Therefore, we studied the colchicine response in FMF patients and its association with the phenotype, disease severity, genotype, and biochemical biomarkers in colchicine-non-responders. Around 41% of patients were colchicine-non-responders, which is in contrast to previous studies reporting significantly lower percentages in other populations [58]. This difference may be attributed to the effect of the unstudied or novel genes, or lack of compliance due to social and economic factors [59].

The colchicine-non-responders in our study suffered from the severe type of the disease, high ESR, anemia, SAA, CRP, renal stones, lower pyrin concentration, and higher methylation level of the MEFV gene exon 2 than colchicine-responders. This agrees with a previous study highlighting that those colchicine-non-responders suffered from severe clinical symptoms with secondary amyloidosis in the French population [59]. In addition, a previous study showed that colchicine treatment is not correlated with MEFV mRNA expression levels [10]. A possible explanation of this observation may be the presence of MEFV-independent genetic modifiers [45,49,53,54,55]. The full-length human MEFV gene is located in the cytoplasm and related with microtubule and actin filaments by the B30.2 domain of pyrin [29]. Alternatively, the spliced form of pyrin is located mainly at the nucleus [15,29]. The MEFV gene is regulated by nonsense-mediated decay, and different isoforms of MEFV transcripts have different cellular localizations and functions in inflammation. Pyrin expression could be upregulated by various cytokines including IFN-γ, TNF-α, and IL-10 [29]. Colchicine everts various anti-inflammatory effects, alters the actin cytoskeleton organization, prevents neutrophil activation, and inhibits pyrin inflammasome activation without affecting its dephosphorylation or release from 14–3-3 proteins [60,61]. 

The disease severity and therapy response differ between patients depending on the *MEFV* genotype, as MEFV gene mutations or the presence of several bacterial toxins caused abnormal pyrin protein production, reduced regulation of inflammasome assembly, resulting in increased inflammation [62]. However, no significant difference was observed between colchicine-responders in terms of genotype. Other studies revealed M694V homozygote FMF patients were colchicine-non-responders [63,64]. This may be due to environmental, genetic, or epigenetic factors [10,12,65].

Our study elucidates that the methylation% and the pyrin concentration may have an impact on FMF pathogenesis. The methylation% may be related to disease severity and renal calculi, but seemed to be independent of the genotyping. Colchicine non-response correlated with the severity of the disease, clinical phenotypes, high ESR, and anemia, but was not associated with methylation or pyrin levels. Nonetheless, limitations in this study include a relatively small number of patients, as we could not address if the pyrin concentration may be used as a biomarker for each studied mutation individually and the relation between colchicine response, genotype, and disease severity. In addition, activation of caspase-1 and IL-1b was not addressed in this study.

## 5. Conclusions

The regulation of MEFV gene expression is a considerably complex process that involves genetic factors coupled with various influences. Further studies involving a larger number of patients are warranted to identify the effects of other epigenetic factors and modifier genes that may affect pyrin concentration and may have an impact on the disease prognosis and phenotype.

## Figures and Tables

**Table 1:** Demographic data of the study population.

Parameter	Patients (n = 63)	Control (n = 19)
Age (years)	4–20	5–20
Male	28 (44.4%)	9 (47.3%)
Female (n %)	35 (55.6%)	10 (52.6%)
Age of onset (mean ± SD)	5.3 ± 3.7	0
Colchicine dose (mg/day) (mean ± SD)	0.5+2.5	0
Consanguineous marriage offspring (n %)	22 (34.9%)	
Similarly affected family members (n %)	54 (85.72%)	-
Colchicine-responders	37 (58.7%)	
Colchicine-non-responders	26 (41.3%)	0

**Table 2:** Relation of MEFV exon 2 methylation% and pyrin concentration with demographic data among FMF patients.

Demographic Data Total n = 63 (100%)	Methylation% (Mean + SD)	*p*-Value	Pyrin Concentration (pg/mL) (Mean + SD)	*p*-Value
Gender:				
Male n = 28 (44.4%)	17.13 ± 12.0	0.268	384.66 ± 73.1	0.994
Female n = 35 (55.6%)	20.41 ± 11.3		384.79 ± 73.7	
Consanguineous marriage:				
Offspring n = 22 (34.9%)	23.95 ± 13.6	0.011 *	391.84 ± 85.1	0.544
Non-offspring n = 41 (65.1%)	16.27 ± 9.5		380.92 ± 56.6	
Affected family members:				
Similarly n = 54 (85.72%)	19.32 ± 11.7	0.539	382.93 ± 70.5	0.609
Not similarly n = 9 (14.3%)	16.73 ± 11.1		395.51 ± 46.8	

*p*-value > 0.05: non-significant; * *p*-value < 0.05: significant.

**Table 3:** Relation of genotypes with MEFV exon 2 methylation% and pyrin concentration in FMF patients.

Genotyping (Mean+ SD)	Methylation %	*p*-Value	Pyrin Concentration (pg/mL)	*p*-Value
Mutations n = 53 (77.8%)	20.2 ± 11.9	0.107	388.27 ± 72.03	0.439
No mutations n = 14 (22.2%)	14.5 ± 9.8		372.30 ± 48.20	
Exon 2 mutations n = 13 (20.6%)	21.6 ± 15.6	0.169	360.0 ± 60	0.244
Exon10 mutations n = 40 (63.5%)	19.8 + 10.5		393.3 ± 73.0	
R202Q n = 2 (3.2%)	25.1 ± 20.8	0.448	322.3 ± 36.8	0.601
M694V n = 4 (6.3%)	24.8 ± 9.3		416.8 ± 60.9	
M694I n = 19 (30.2%)	21.5 ± 11.9		387.2 ± 88.8	
E148Q n = 11 (17.5%)	20.9 ± 15.7		366.9 ± 63.1	
V726A n = 6 (9.5%)	18.9 ± 7.9		399.5 ± 52.1	
M680I n = 13 (20.6%)	18.9 ± 7.9		395.6 ± 56.7	
Heterozygous n = 34 (53.96%)	19.29 ± 12.06		386.54 ± 64.2	
Homozygous n = 8 (12.6%)	23.038 ± 12.06	0.466	413.07 ± 112.2	0.469
Compound heterozygous n = 7 (5.55%)	22.52 ± 15.44		354.53 ± 42.965	

*p*-value > 0.05: non-significant.

**Table 4:** Relation of MEFV exon 2 methylation% and pyrin concentration with disease severity among patients.

Parameters/ Disease Severity Total n = 63 (100%)	Methylation% (Mean + SD)	*p*-Value	Pyrin Concentration (pg/mL) (Mean + SD)	*p*-Value
Mild n = 13 (20.6%)	11.36 ± 7.8	0.016 *	392.06 ± 51.2	0.664
Moderate and severe n = 50 (79.3%)	20.4 ± 12.1		382.82 ± 71.4	

*p*-value > 0.05: non- significant; * *p*-value < 0.05: significant.

**Table 5:** Disease severity and genotyping.

	Severe n = 32 (50.8%)	Moderate n = 18 (28.6%)	Mild n = 13 (20.6%)	*p*-Value
Mutations n = 49 (77.8%)	26 (81.25%)	14 (77.7%)	9 (69.23%)	0.68
No mutations n = 14 (22.2%)	6 (18.75%)	4 (22.22%)	4 (30.7%)	
Exon 2 mutations n = 13 (20.6)	7 (53.8%)	2 (15.4%)	4 (30.8%)	0.31
Exon 10 mutations n = 40 (63.5)	21 (52.5%)	13 (32.5%)	6 (15.0%)	
E148Q n = 11 (17.5)	5 (15.5%)	2 (10.0%)	4 (28.5%)	0.267
R202Q n = 2 (3.2)	2 (6.0%)	0	0	
M694I n = 19(30.2	12 (63.2%)	5 (25.0%)	2 (14.25%)	
M680I n = 13 (20.6)	5 (15.15%)	6 (30.0%)	2 (14.2%)	
V726A = 6 (9.5)	1 (3.0%)	3 (15.0%)	2 (14.2%)	
M694V n = 4 (6.3)	4 (12.12%)	0	0	

*p*-value > 0.05: non-significant.

**Table 6:** Correlation of the methylation % and pyrin concentration with the phenotype of patients.

Phenotype (n%) (Mean + SD)	Methylation%	Pyrin Concentration (pg/mL)	r	*p*-Value
Abdominal pain n = 45 (71.4%)	19.91 ± 12.1	380.91 ± 63.8	−0.084	0.582
Arthritis n = 41 (65.1%)	19.8 ± 11.7	373.49 ± 62.0	−0.095	0.554
Fever n = 37 (58.7%)	18.64 ± 11.2	383.81 ± 63.8	−0.021	0.904
Diarrhea/constipation n = 25 (39.7%)	20.43 ± 13.7	370.46 ± 50.0	−0.056	0.791
Erysipeloid erythema n = 12 (19.0%)	22.19 ± 12.2	377.2 ± 53.5	0.01	0.976
Vomiting n = 10 (15.9%)	25.27 ± 12.8	390.1 ± 31.3	0.576	0.082
Chest pain n = 6 (9.5%)	17.05 ± 6.4	441.57 ± 45.9	−0.087	0.869

*p*-value > 0.05: non-significant.

**Table 7:** Relation of the colchicine response with different parameters in FMF patients.

Parameters	Non-Responder Patients n = 26 (41.3%)	Responder Patients n = 37 (58.7%)	*p*-Value
Methylation%	19.54 ± 11.7	18.53 ± 11.7	0.737
Pyrin concentration (pg/mL)	378.0 ± 62.4	389.5 ± 71.2	0.512
Abdominal pain n = 45 (71.4%)	26 (100.0%)	19 (51.4%)	<0.001 **
Arthritis n = 41 (65.1%)	25 (69.2%)	16 (43.2%)	<0.001 **
Fever n = 37 (58.7)	20 (76.95%)	17 (45.9%)	0.014 *
Erysipeloid erythema n = 12 (19.0%)	9 (34.6%)	3 (8.1%)	0.008 **
Vomiting n = 10 (15.9%)	6 (23.1%)	4 (10.8%)	0.19
Diarrhea/constipation n = 25 (39.7%)	15 (57.7%)	10 (27.0%)	0.014 *
Chest pain n = 6 (9.5%)	5 (19.2%)	1 (2.7%)	0.028 *
FMF severity			<0.001 **
Mild n = 13 (20.6)	0	13 (35.1%)	
Moderate n = 18 (28.6)	4 (15.4%)	14 (37.8%)	
Severe n = 32 (50.8)	22 (84.6%)	10 (27.0%)	
Homozygous n = 8 (12.6%)	4 (50.0%)	4 (50.0%)	0.628
Compound heterozygous n = 7 (5.55%)	4(70.0%)	3(30.0%)	
Heterozygous n = 34 (53.96%)	13 (38.2%)	21(61.8%)	
High SAA n = 13 (20.6%)	8 (30.8%)	5 (13.5%)	0.09
High CRP n = 21 (33.3%)	12 (46.2%)	9 (24.3%)	0.07
High ESR n = 28 (44.4%)	16 (61.5%)	12 (32.4%)	0.022 *
Anemia n = 32 (50.8%)	17 (65.4%)	15 (40.5%)	0.05 *
H-pylori n = 8 (12.7%)	4 (15.4%)	4 (10.8%)	0.591
Renal calculi n = 14 (22.2%)	8 (30.8%)	6 (16.2%)	0.171
Vitamin D deficiency n = 24 (31.7%)	13 (50.0%)	11 (29.7%)	0.103

*p*-value > 0.05: non-significant; * *p*-value < 0.05: significant; ** *p*-value < 0.01: highly significant; ESR: erythrocyte sedimentation rate; CRP: C-reactive protein; SAA: serum Amyloid A; H-pylori: Helicobacter pylori.

## Data Availability

Not applicable.

## References

[1] Tan S., Zhang J., Tee W. (2022). Epigenetic Regulation of Inflammatory Signaling and Inflammation-Induced Cancer. Front. Cell Dev. Biol..

[2] Maximilian H., Giacomo C. (2022). Molecular Mechanisms of Trans-generational Epigenetic Inheritance. Nat. Rev. Genet..

[3] Alyson A., Vincent C., Benjamin P. (2020). How does epigenetics influence the course of evolution?. Philos. Trans. R. Soc. B.

[4] Tuong Z., Stewart B., Guo S., Clatworthy M. (2022). Epigenetics and Tissue Immunity—Translating Environmental Cues into Functional Adaptations. Immunol. Rev..

[5] Álvarez-Errico D., Vento-Tormo R., Ballestar E. (2017). Genetic and Epigenetic Determinants in Auto-inflammatory Diseases. Front. Immunol..

[6] Saxena R., Tiwari K. (2022). Role of Epigenetics in Developing Therapeutic Strategies against COVID-19. J. Clin. Diagn. Res..

[7] Santaló J., Berdasc M. (2022). Ethical Implications of Epigenetic in the Era of Personalized Medicine. Clin. Epigenetics.

[8] Li J., Li L., Wang Y., Huang G., Li X., Xie Z., Zhou Z. (2021). Insights Into the Role of DNA Methylation in Immune Cell Development and Autoimmune Disease. Front. Cell Dev. Biol..

[9] Pasquale S., Assunta S., Cesar A., Giorgio G., Giovanni N., Francesca R., Roberta T., Claudio S. (2020). Metabolic Regulation of Epigenetic Modifications and Cell Differentiation in Cancer. Cancers.

[10] Dogan E., Gursoy S., Bozkaya G., Camlar S., Kilicarslan O., Soylu A., Ulgenalp A., Kavukcu S., Bozkaya O.G. (2019). The Effects of Epigenetic Regulation on Phenotypic Expressivity in Turkish Patients with Familial Mediterranean fever. Indian J. Rheumatol..

[11] Sebastian K., Silvia D., Christiane W., Michael B., Sevi D., Lukas B., Dirk S. (2022). Evidence that direct inhibition of transcription factor binding is the prevailing mode of gene and repeat repression by DNA methylation. Nat. Genet..

[12] Yutaka M., Izuru O., Erik W., Daichi M., Kenji S., Masahiro S. (2022). Structural Insights into Methylated DNA Recognition by the MethylCpG Binding Domain of MBD6 from Arabidopsis thaliana. ACS Omega.

[13] Sammallahti S., Koopman-Verhoef M., Binter A., Mulder R., Cabré-Riera A., Kvist T., Malmberg A., Pesce G., Plancoulaine S., Heiss J. (2022). Longitudinal Associations of DNA Methylation and Sleep in Children: A meta-analysis. Clin. Epigenetics.

[14] Zelin J., Yun L. (2018). DNA methylation in human diseases. Genes Dis..

[15] Erdem G., Erdemir S., Abaci I., Kirectepe A., Everest E., Turanli E. (2017). Alternatively spliced MEFV transcript lacking exon 2 and its protein isoform pyrin-2d implies an epigenetic regulation of the gene in inflammatory cell culture models. Genet. Mol. Biol..

[16] Carlos D., Octavio M., Esteban B. (2020). Understanding the Relevance of DNA Methylation Changes in Immune Differentiation and Disease. Genes.

[17] Krista S., Thomas P., Robert J., Lynn B. (2012). Folate and DNA Methylation: A Review of Molecular Mechanisms and the Evidence for Folate’s Role. Am. Soc. Nutrition. Adv. Nutr..

[18] Funes S., Fernández-Fierro A., Rebolledo-Zelada D., Mackern-Oberti J., Kalergis A. (2021). Contribution of Dysregulated DNA Methylation to Autoimmunity. Int. J. Mol. Sci..

[19] Migita K., Asano T., Sato S., Koga T., Fujita Y., Kawakami A. (2018). Familial Mediterranean fever: Overview of Pathogenesis, Clinical Features and Management. Immunol. Med..

[20] Mansour A., El-Shayeb A., ElHabachi N., Khodair M., Elwazzan D., Abdeen N., Said M., Ebaid R., El-Shahawy N., Seif A. (2019). Molecular Patterns of *MEFV* Gene Mutations in Egyptian Patients with Familial Mediterranean Fever: A Retrospective Cohort Study. Int. J. Inflamm..

[21] Mansour A. (2017). Familial Mediterranean fever, review of the literature. Clin. Rheumatol..

[22] Abukhalaf S., Dandis B., Tari T., Alzughayyar A., Rajabi Y. (2020). Familial Mediterranean fever Complicated by a Triad of Adrenal Crisis: Amyloid Goiter and Cardiac Amyloidosis. Case Rep. Rheumatol..

[23] Ozen S., Kone-Paut I., Gül A. (2017). Colchicine Resistance and Intolerance in Familial Mediterranean fever: Definition, Causes, and Alternative Treatments. Semin. Arthritis Rheum..

[24] Maggio M., Corsello G. (2020). FMF is not always “fever”: From clinical presentation to “treat to target”. Ital. J. Pediatr..

[25] Manukyan G., Aminov R. (2016). Update on Pyrin Functions and Mechanisms of Familial Mediterranean fever. Front. Microbiol..

[26] Yvan J., Lucie L., Flora M., Amandine M., Sarah B., Omran A., Mathilde P., Ve’ronique H., Pascal S., Mathieu G. (2018). Familial Mediterranean fever mutations are hypermorphic mutations that specifically decrease the activation threshold of the Pyrin inflammasome. Rheumatology.

[27] Mangan M., Gorki F., Krause K., Heinz A., Pankow A., Ebert T. (2020). Transcriptional licensing is required for Pyrin inflammasome activation in human macrophages and bypassed by mutations causing familial Mediterranean fever. PLoS Biol..

[28] Heilig R., Petr B. (2018). Function and Mechanism of the Pyrin Inflammasome. Eur. J. Immunol..

[29] Schnappauf O., Chae J., Kastner D., Aksentijevich I. (2019). The PyrinInflammasome in Health and Disease. Front. Immunol..

[30] Sei S., Ryusuke Y., Yohei K., Hideaki N. (2021). The PRY/SPRY domain of pyrin/TRIM20 interacts with β2-microglobulin to promote infammasome formation. Sci. Rep..

[31] Sevinç N., Erdem G., Kireçtepe-Aydın A., Altin S. Increased Pyrin Protein Levels in Patients with Familial Mediterranean Fever (FMF) could be Independent of Mediterranean Fever Gene (MEFV) Exon10 Variations. Proceedings of the 12th National Congress of Medical Genetics Conference, Turkish Society of Medical Genetics.

[32] Kirectepe A., Kasapcopur O., Arisoy N., Erdem G., Hatemi G., Ozdogan H. (2011). Analysis of MEFV Exon Methylation and Expression Patterns in Familial Mediterranean Fever. BMC Med. Genet..

[33] Samli H., Dogru O., Bukulmez A., Yuksel E., Ovali F., Solak M. (2006). Relationship of Tel Hashomer Criteria and Mediterranean fever Gene Mutations in a Cohort of Turkish Familial Mediterranean Fever Patients. Saudi Med. J..

[34] Pras E., Livneh A., Balow J.E., Pras E., Kastner D.L., Pras M., Langevitz P. (1998). Clinical Differences between North African and Iraqi Jews with Familial Mediterranean fever. Am. J. Med. Genet..

[35] Babidge W., Butler L., Burton M., Cowled P. (2001). Methylation of CpG Sites in Exon 2 of the Bcl-2 Gene occurs in Colo-rectal Carcinoma. Anticancer Res..

[36] Lo P.K., Watanabe H., Cheng P.C., Teo W.W., Liang X., Argani P., Lee J.S., Sukumar S. (2009). Methy SYBR, a Novel Quantitative PCR Assay for the Dual Analysis of DNA Methylation and CpG Methylation Density. J. Mol. Diagn..

[37] Al-Haggar M., Yahia S., bdel-Hady D., Al-Saied A., Al-Kenawy R., Abo-El-Kasem R. (2014). Phenotype-Genotype Updates from Familial Mediterranean Fever Database Registry of Mansoura University Children Hospital, Mansoura, Egypt. Indian J. Hum. Genet..

[38] Talaat H.S., Sheba M.F., Mohammed R.H., Gomaa M.A., El Rifaei N., Ibrahim M.F.M. (2020). Genotype Mutations in Egyptian Children with Familial Mediterranean fever: Clinical Profile, and Response to Colchicines. Mediterr. J. Rheumatol..

[39] El Gezery D., Abou-Zeid A., Hashad D., El-Sayegh H. (2010). MEFV Gene Mutations in Egyptian Patients with Familial Mediterranean fever. Genet. Test. Mol. Biomark..

[40] Yomna F., Heba T., Noha M., Diana F., Huda M. (2020). Articular manifestations in Egyptian children with familial Mediterranean fever. Egypt. Rheumatol. Rehabil..

[41] Lofty H.M., Marzouk H., Farag Y., Nabih M., Khalifa I.A., Mostafa N., Salah A., Rashed L., El Garf K. (2016). Serum Amyloid A level in Egyptian Children with Familial Mediterranean Fever. Int. J. Rheumatol..

[42] Mohammed H., Omar E., Eslam M., Aya M. (2022). Clinical and genetic characterization of familial Mediterranean fever among a cohort of Egyptian patients. Gastroenterol. Rev..

[43] Beshlawy A., Zekri A., Ramadan M., Selim Y., Abdel-Salam A., Hegazy M., Ragab L., Gaggiano C., Cantarini L., Ragab G. (2022). Genotype-phenotype associations in familial Mediterranean fever: A study of 500 Egyptian pediatric patients. Clin. Rheumatol..

[44] Taryma-Leśniak O., EwaSokolowska K., Kazimierz T. (2020). Current Status of Development of Methylation Biomarkers for in Vitro Diagnostic IVD Applications. Clin. Epigenetics.

[45] Turanli E., Kirectepe A., Kasapçopur Ö. (2013). MEFV Methylation Analysis in FMF, and JRA Diseases. Pediatr. Rheumatol..

[46] Yepiskoposyan L., Harutyunyan A. (2007). Population genetics of familial Mediterranean fever: A review. Eur. J. Hum. Genet..

[47] Anderson O., Sant E., Dolinoy D. (2012). Nutrition and epigenetics: An interplay of dietary methyl donors, one-carbon metabolism and DNA methylation. J. Nutr. Biochem..

[48] Kandi V., Vadakedath S. (2015). Effect of DNA methylation in various diseases and the probable protective role of nutrition: A mini-review. Cureus.

[49] Sharkia R., Mahajnah M., Zalan A., Athamna M., Azem A., Badarneh K., Faris F. (2013). Comparative Screening of FMF Mutations in Various Communities of the Israeli Society. Eur. J. Med. Genet..

[50] Shahbaznejad L., Raeeskarami S.R., Assari R., Shakoori A., Azhideh H., Aghighi Y., Tahghighi F., Ziaee V. (2018). Familial Mediterranean Gene (MEFV) Mutation in Parents of Children with Familial Mediterranean fever: What Are the Exceptions?. Int. J. Inflamm..

[51] El-Messery L., Elhagrasy H. (2014). Study of MEFV Gene R202Q Polymorphism in Egyptian Patients with Familial Mediterranean fever. Egypt. J. Haematol..

[52] Kehribar D., Özgen M. (2020). The Importance of Mediterranean fever Gene in Familial Mediterranean Fever. Eur. J. Rheumatol..

[53] Ben-Zvi I., Livneh A. (2010). Chronic Inflammation in FMF: Markers, Risk Factors, Outcomes and Therapy. Nat. Rev. Rheumatol..

[54] Iwona G., Mariusz K., Andrzej C., Klaudyna L., Paweł J., Oksana B., Bartosz K., Andrzej B., Lech C. (2019). Polymorphism of Interleukin 1B May Modulate the Risk of Ischemic Stroke in Polish Patients. Medicina.

[55] Panel C., Hasmik A., Stéphanie P. (2000). Identification of MEFV independent Modifying Genetic factors for FMF. Am. J. Hum. Genet..

[56] Duşunsel R., Dursun I., Gündüz Z., Poyrazoğlu M., Gürgöze M., Dundar M. (2008). Genotype–Phenotype correlation in children with familial Mediterranean fever in a Turkish population. Pediatr. Int..

[57] Liu J., Morgan M., Hutchison K., Calhoun V. (2010). A Study of the Influence of Sex on Genome Wide Methylation. PLoS ONE.

[58] Corsia A., Georgin-Lavialle S., Hentgen V., Hachulla E., Grateau G., Faye A., Quartier P., Rossi-Semerano L., Koné-Paut I. (2017). A Survey of Resistance to Colchicine Treatment for French Patients with Familial Mediterranean fever. Orphanet J. Rare Dis..

[59] Lidar M., Scherrmann J.M., Shinar Y., Chetrit A., Niel E., Gershoni-Baruch R., Langevitz P., Livneh A. (2004). Colchicine Non-responsiveness in Familial Mediterranean fever: Clinical, Genetic, Pharmacokinetic, and Socioeconomic Characterization. Semin. Arthritis Rheum..

[60] Gao W., Yang J., Liu W., Wang Y., Shao F. (2016). Site-Specific Phosphorylation and Microtubule Dynamics Control Pyrin Inflammasome Activation. Proc. Natl. Acad. Sci. USA.

[61] Ying Y., Laura L., Virginia B. (2015). Colchicine—Update on mechanisms of action and therapeutic uses. Semin. Arthritis Rheum..

[62] Mansfield E., Chae J.J., Komarow H.D., Brotz T.M., Frucht D.M., Aksentijevich I., Kastner D.L. (2001). The Familial Mediterranean Fever Protein, Pyrin, Associates with Microtubules and Colocalizes with Actin Filaments. Blood.

[63] Soylemezoglu O., Arga M., Fidan K., Gonen S., Emeksiz H., Hasanoglu E., Buyan N. (2010). Unresponsiveness to colchicine therapy in patients with familial Mediterranean fever homozygous for the M694V mutation. J. Rheumatol..

[64] Lidar M., Yonath H., Shechter N., Sikron F., Sadetzki S., Livneh A., Pras E. (2012). Incomplete response to colchicine in M694V homozygote FMF patients. Autoimmun. Rev..

[65] Mohamed A., Rania A., Eman B. (2021). Genotype Phenotype Correlation of FMF Cases in East Delta of Egypt. Egypt. J. Hosp. Med..

